# Selection Acts on DNA Secondary Structures to Decrease Transcriptional Mutagenesis

**DOI:** 10.1371/journal.pgen.0020176

**Published:** 2006-11-03

**Authors:** Claire Hoede, Erick Denamur, Olivier Tenaillon

**Affiliations:** INSERM U722 and Université Paris 7—Denis Diderot, Faculté de Médecine, Site Xavier Bichat, Paris, France; National Institute of Genetics, Japan

## Abstract

Single-stranded DNA is more subject to mutation than double stranded. During transcription, DNA is transiently single stranded and therefore subject to higher mutagenesis. However, if local intra-strand secondary structures are formed, some bases will be paired and therefore less sensitive to mutation than unpaired bases. Using complete genome sequences of *Escherichia coli,* we show that local intra-strand secondary structures can, as a consequence, be used to define an index of transcription-driven mutability. At gene level, we show that natural selection has favoured a reduced transcription-driven mutagenesis via the higher than expected frequency of occurrence of intra-strand secondary structures. Such selection is stronger in highly expressed genes and suggests a sequence-dependent way to control mutation rates and a novel form of selection affecting the evolution of synonymous mutations.

## Synopsis

Genome sequence evolution results from the interplay between mutagenesis and natural selection. Mutations occur as the result of biochemical or physical alteration of DNA and/or from the errors made by polymerases while replicating DNA. As many mutations tend to be detrimental to the organism's fitness, natural selection favours a decrease in mutation rate. Hence, many mechanisms have evolved to control mutation rate. The mechanisms described to date have relied on (i) the existence of enzymes repairing the damaged DNA or correcting mismatched bases, which are mechanisms having an effect on whole genome mutation rate, and (ii) the avoidance in the sequence of repetition that could be misread by the polymerases, which is a sequence-dependent local control of mutation rate. In the present paper, the authors suggest that another sequence-dependent control of mutation exists and shapes the overall evolution of the genome. Using a comparative analysis of Escherichia coli genomes, they show that local secondary structures that are formed during the transcription of genes into RNA can modulate the base-to-base mutation rate. Moreover, the authors show that natural selection seems to have favoured the occurrence of such structures to minimise mutability, especially in the most expressed genes. This paper proposes a new way in which gene sequences can be constrained by natural selection.

## Introduction

DNA mutation results from a combination of chemical alterations of nucleotides and base mis-incorporation (stochastic or damage-driven) by DNA polymerases [1]. The spontaneous rate of mutation is not homogenous along the whole chromosome. Base composition and sequence-specific biases in mutation rates exist, e.g., tracts of guanine facilitate polymerase slippage [1], and mutations from GC to AT base pairs tend to be prevalent [2]. Temporal variations in mutation rate also exist: while being transcribed, DNA is transiently single stranded and its bases are therefore much more sensitive to chemical alterations [3,4]. Recently, temporal and sequence-specific variation in the mutation rate has been shown in some bacterial genes [5] and in the human cancer-linked gene *P53* [6]. The mechanism proposed to explain these observations is the following: during transcription, while DNA is single stranded, local intra-strand secondary structures are transiently created, depending upon the nucleotide sequence; bases paired in such structures are more protected from alterations than unpaired bases. Hence, both transcription-level and local sequence composition modulate the spontaneous rate of transcription-driven mutagenesis (TDM). Based on the stability of local DNA secondary structure, it is possible to assign each nucleotide a mutability index that has some predictive power on its spontaneous mutation rate [4]. Using experimental data from *Escherichia coli,* Wright et al. showed in pioneering work that such an index can be calculated by folding 30-bp subsequences [5].

As a large fraction of mutations tend to be deleterious [7,8], a plethora of DNA repair mechanisms have been selected for, and the parallel action of these mechanisms can result in per base mutation rates as low as 10^−9^ per generation [1,9]. Most of these mechanisms act through enzymes that correct damaged or erroneously incorporated bases over the whole chromosome. However, as TDM varies with gene transcription level and local sequence composition, it offers a means by which mutation rates can be modulated locally through a preventive mechanism, as opposed to enzymatic mechanisms that act globally and rely on the identification of errors after they have already occurred. Gene expression level has already been shown to impact genome evolution as it modulates synonymous mutation [10–12] and amino acid substitution rates [13,14]; in the present paper we investigate the influence of TDM on genome evolution and show that the control of TDM through DNA secondary structures is under selection in the E. coli bacterial genome.

## Results/Discussion

### A Transcription-Driven Mutability Index Based on Relative Time Spent Unpaired

We defined a transcription-driven mutability index (TDMI) that, averaged over an entire gene, would reflect the overall mutability of that gene. We suspected that there would be a correlation between TDM and the time spent by bases in an unpaired state, and thus defined the TDMI as the relative amount of time spent by a base in an unpaired state. We proceeded as follows: (i) all 30-bp subsequences including a given base, *x*, were folded using the program hybrid-ss-min in the software OligoArrayAux 1.9 [15,16]; (ii) for each subsequence, both the free energy (Δ*G*) of its most stable structure and the paired/unpaired state of base *x* in this fold were recorded; and (iii) the TDMI of base *x* was calculated as the ratio of the sum of exp(−Δ*G*/*RT*) over all most-stable folds in which *x* was unpaired and the sum of exp(−Δ*G*/*RT*) over all most-stable folds that include base *x* (in which *T* is the temperature in degrees Kelvin and *R* is the perfect gas constant). TDMI is thus constrained to lie between zero and one, with one corresponding to a higher chance of being affected by TDM.

### Variable Bases in Genome Alignment Present a Higher TDMI

**Figure 1:**
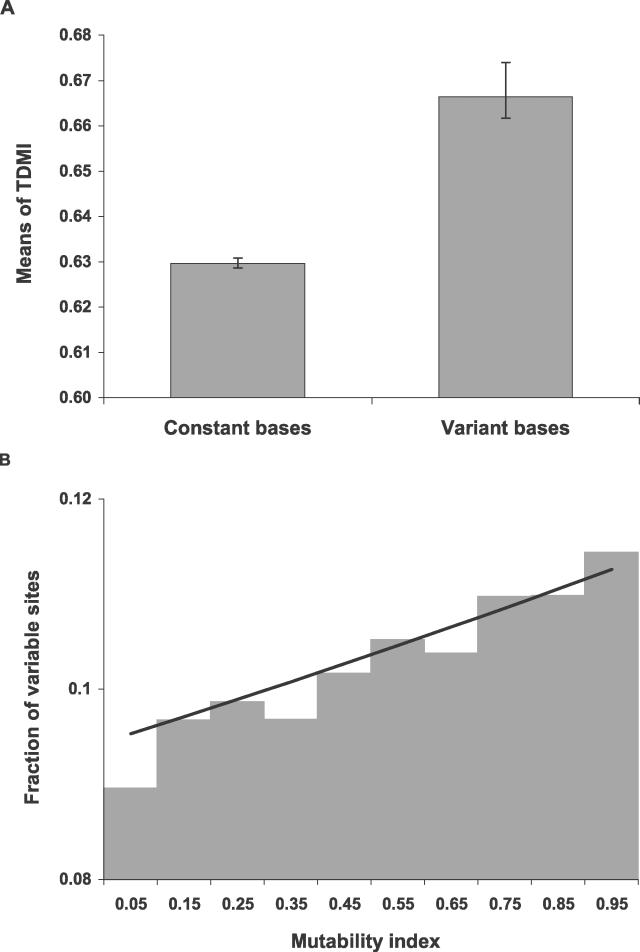
Relationship between TDMIs of L4 Sites and Their Variation during E. coli MG1655 and E. coli CFT073 Divergence (A) This graph represents average TDMI at constant and variable L4 sites in gene by gene alignment of the completely sequenced strains E. coli CFT073 and E. coli MG1655. In both genomic contexts, averages of TDMI of constant sites are exactly the same: 0.630. For the variable sites, averages of TDMI are 0.668 for MG1655 and 0.665 for CFT073. The error bars are 95% CIs on the mean estimated by bootstrap. (B) This graph represents the fraction of variable L4 sites as a function of TDMI (histogram) and logistic regression (*p* < 2 × 10^−16^, *n* = 550,575) (line).

We compared the TDMI of bases that varied among three fully sequenced E. coli genomes to those that remained constant. To avoid strong selection effects that might obscure the signal, we restricted our analysis to 4-fold degenerate sites (L4 sites); as mutations at these sites do not affect the protein sequence, they are least affected by selection. We used pairwise nucleotide alignments of orthologous genes in three genomes of *E. coli:* MG1655 [17], CFT073 [18], and O157:H7 [19]. As shown in Figure 1A, each pairwise comparison revealed that variable sites had a higher TDMI than constant ones (*n* = 550,575, two-tailed *t*-test, *p* < 2.2 × 10^−16^, randomisation test, *p* < 2.2 × 10^−16^), as expected by our model of mutagenesis. Our model also suggests that the relative importance of TDM should be an increasing function of expression level, which can be approximated by major codon usage (MCU), a measure of codon bias [20] that reflects the intensity of natural selection acting on synonymous codons to enhance translation fidelity and efficiency. Indeed, since 1981, it has been highlighted that gene expression level influences the choice of synonymous codons, with codons having more tRNA being preferentially used in highly expressed genes [10–12]. Using logistic regressions on the previous dataset, we estimated that the probability of each L4 site changing between CFT073 and MG1655 was increased by 20.7% when TDMI increased from zero to one (Figure 1B). We performed the same analysis on a subset of genes characterised by their MCU. For genes with MCU ranging from 0.4 to 0.6 (low level of expression), 0.6 to 0.7 (high level of expression), and higher than 0.7 (very high level of expression), the relative impact of TDMI was, respectively, 17.5% (95% confidence interval [CI]: 0.156–0.190), 29.6% (95% CI: 0.254–0.332), and 14.4% (95% CI: 0.080–0.210). Hence, expression level increased the impact of TDM as expected, but in very highly expressed genes the intensity of selection acting on synonymous codons through codon bias became substantial and obscured the signal of mutagenesis so that the observed variability between strains at L4 sites was a mixture of mutation and selection. These observations validated our model of mutagenesis and the use of TDMI as an indicator of mutability; more importantly, they suggested that the modulation of TDM through selection might be strong enough to leave a distinctive footprint in the genome.

### Gene Average TDMI Is Influenced by GC Content

**Figure 2:**
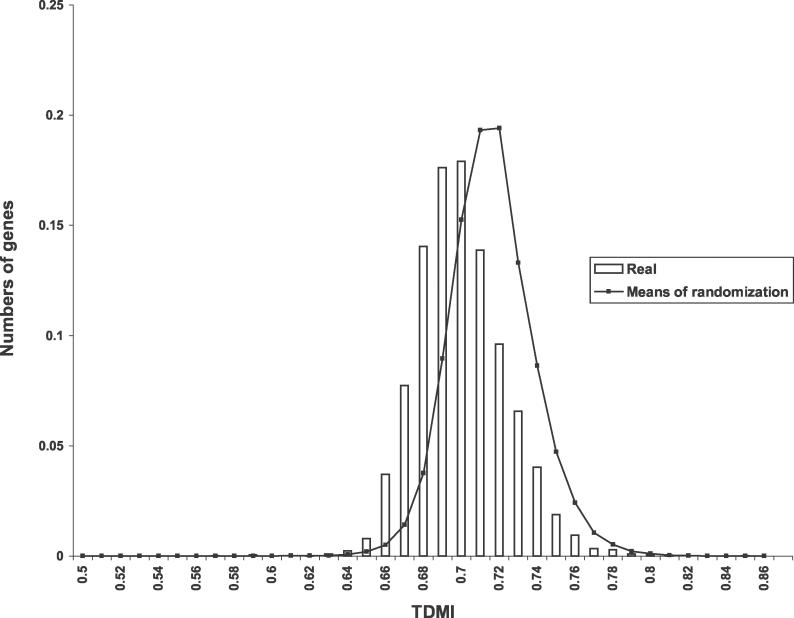
Distributions of Gene Average TDMI for E. coli MG1655 Genome and for Five Virtual Genomes The open bars show the distribution for the MG1655 genome; the line shows the distribution for five virtual genomes composed of shuffled genes with identical length and GC% as the observed genes. The E. coli genome has a lower gene TDMI than expected from nucleotide composition (Student's two-tailed *t-*test *p* < 2.2 × 10^−16^).

To detect if selection had influenced the values of TDMI observed in bacterial genomes, we compared the observed TDMI values with the values obtained using various randomisation processes on the genome. These randomisations aimed at producing the TDMI patterns expected by chance. We first studied the distribution of gene TDMI, i.e., average TDMI values of all the bases of the gene, of the E. coli MG1655 strain. We compared this distribution to the one obtained in randomised genomes, i.e., genomes having the same number of genes and in which gene length and GC percent content (GC%) are identical to those of the observed genome, but in which each gene has its nucleotide sequence shuffled. As presented in Figure 2, the observed distribution was significantly skewed towards lower gene TDMI (two-tailed *t*-test, *p* < 2.2 × 10^−16^), a pattern reflecting selection to minimise mutability at the genome scale.

To better understand the determinants of such selection, we then contrasted gene TDMI with various gene features. Gene TDMI correlated negatively with GC%, gene expression, and MCU. Whereas TDMI correlated poorly with GC% (*r* = −0.23) when evaluated on random genomes, the correlation with GC% was very strong on the observed genome (*r* = −0.7), suggesting that high GC% genes had evolved the lowest mutability. The negative correlation with MCU (*p* = 4.46 × 10^−11^ in multiple regression including GC%, adjusted *r*-squared: 0.4919) reflected that the highly expressed genes tended to have lower TDMI. However, the impact of MCU on the quality of the multiple regression was marginal compared to the impact of GC% (adjusted *r*-squared: 0.4887 and 0.4919 for GC% and GC% + MCU, respectively). Several factors could explain why GC-rich genes tend to evolve lower TDMI. As GC bases are more sensitive to TDM [2,5], selection to minimise TDM could be stronger in GC-rich genes. Alternatively, as GC pairing is stronger than that of AT, it is possible that selection acting to minimise TDMI can be achieved more easily in GC-rich genes that allow more stable secondary structures to be made.

### High Gene Expression Selects for an Effective Decrease in Intrinsic TDMI

**Figure 3:**
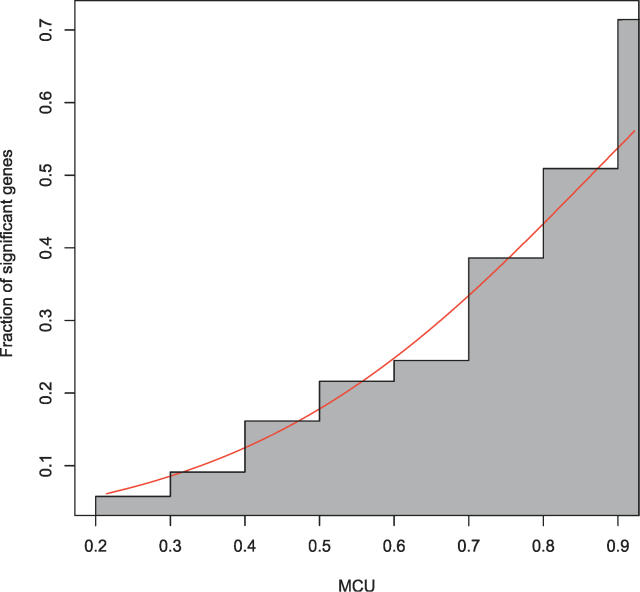
Fraction of Significant Genes Predicted from MCU by Logistic Regression Logistic regression (*p* < 2.2 × 10^−16^) linking the fraction of genes significantly robust (i.e., being significantly less mutable than expected according to protein sequence) to gene MCU (used here as a proxy for gene expression). The red line represents the fraction of significant genes predicted by the regression, and the grey histogram represents the observed fraction of significant genes by class of gene MCU.

One of the limits of the previous approach is that it does not consider the constraints imposed by gene function on amino acid sequence and subsequently on DNA sequence. A specific function could require a protein whose amino acids have AT-rich codons. Constraints imposed by gene function could limit the minimum value gene TDMI could reach. This could erase the footprint of selection acting to minimise TDMI, as some constrained genes in which TDMI has been minimised could have higher TDMI than less constrained genes. In order to investigate these effects, we undertook an alternative approach in which we compared the observed TDMI to that expected when genes were randomised by having their synonymous codons shuffled. All randomised genes thus code for the same proteins and share the same GC% and same codon bias as the observed genes. A gene was considered intrinsically robust to mutation if its TDMI was lower than the TDMI of 95% of this randomised set of genes. All 4,307 genes were randomised 1,000 times, an operation that required folding several billion subsequences. Over 20% of genes were identified as robust (5% expected by chance alone), revealing that selection was acting on many genes to reduce the TDMI. Using logistic multiple regressions, we identified MCU (Figure 3) as the only predictive factor of robust genes (logistic regression between MCU, GC%, and fraction of significant gene: *p*(MCU) < 2 × 10^−16^, *p*(GC%) = 0.131). The genes having the highest MCU had a 55% chance of being robust to mutation (i.e., of having a TDMI lower than 95% of the randomised set of genes). This result was consistent with the fact that TDM increases with expression level and could subsequently result in stronger selection for reduced TDMI. Essential genes [21] and old genes (genes introduced long ago into the chromosome) [22] were more robust than others. However, the significance of these factors disappeared when MCU was also taken into account in the analysis, revealing that expression intensity is the key factor that drives selection for reduced TDMI. Along the same lines, gene function classes associated with strong expression tended to contain more robust genes.

### Selection for Lower TDM rather than mRNA Stability

Because we are studying DNA intra-strand secondary structures, one could argue that some form of selection acting at the RNA level is responsible for our observations. Two points lead us to think that selection for reduced TDMI at the DNA level is relevant: (i) high TDMI is associated with increased mutation rate at the DNA level, as evidenced by comparison of orthologous genes (Figure 1A); and (ii) to our knowledge there is no biological evidence of a general link existing between RNA stability and secondary structure in the coding regions of prokaryotes (but see for eukaryotes [23]), although some special cases have been described [24]. Using published experimental estimations of mRNA half-life [25], we could not identify any correlation of mRNA half-life with either TDMI or the probability of being robust. Some previous bioinformatics studies on enterobacteria have found an excess of secondary structure in the genome within coding sequences [26–29] using whole gene length or, as we do, short subsequences of genes. Whereas most studies suggested a link between secondary structures and RNA stability based on verbal arguments, one study [28] supported such a link based on the observation that genes in operons increased in stability as they approached the 3′ end of the mRNA (the end that gets digested first by RNAses). We reproduced their analysis on a larger sample of operons that were five genes long and could not identify any effect of gene position in operons on their stability statistics nor on ours (unpublished data). This suggests that this effect might have been due to limited sampling or to differences in gene features among operons of various lengths.

In the present paper, we suggest that selection might have acted on DNA sequences to decrease the probability of mutation during transcription. Such selection for controlled mutation rate increases with the expression level of genes. More than 50% of highly expressed genes present a non-random combination of synonymous codons that results in lower overall gene mutability. Such TDMI optimisation produces a positioning bias in synonymous codons, demonstrating another non-neutral usage of synonymous codons that could contribute to the reduced synonymous substitution rate observed in highly expressed genes. As the most highly expressed genes did not present the lowest gene mutability, we hypothesise that the strength of selection acting to minimise TDM is as weak or even weaker than the one acting on codon bias [30,31]. It is likely that in highly expressed genes, the two forces compete against one another, a factor that could help explain why these genes do not have the optimal codon usage [32]. As TDMI optimisation involves DNA secondary structures using both synonymous and non-synonymous sites, it could also contribute to the observed reduction in non-synonymous substitution rate associated with higher gene expression [13,14].

We performed the same analysis to the Buchnera aphidicola strain APS genome [33], a genome close to E. coli but in which selection is thought to have been relaxed because of an obligate intracellular lifestyle and reduced population sizes [34]. As would be expected if selection to minimise TDM is weak, we could not find any trace of such selection in the B. aphidicola strain APS genome. Therefore, not all bacterial genomes necessarily present the patterns described in the present study, and it will be of great interest to investigate how selection for reduced TDM is spread across other bacterial species.

## Materials and Methods

### Genomes used.

For this study, we used the fully sequenced *E. coli* genomes of strains MG1655 [17], CFT073 [18], and O157:H7 [19], and a genome of another bacteria, B. aphidicola strain APS [33], which is an endocellular symbiont harboured by pea aphids.

### Determination of TDMI.

To determine the bases that are protected or not during transcription, we extracted all the annotated genes (except the pseudogenes) of genomes. Then we carried out folding of each subsequence of 30 nucleotides, using the program hybrid-ss-min in the software OligoArrayAux 1.9. This program folds single-strand DNA and measures energies for Watson-Crick (A/T and G/C) and wobble pairs (G/T) [15,16].

Wright et al. [5] defined a mutability index (MI) as follows: (total percentage of foldings in which the base is unpaired) × (the lowest −ΔG of the foldings in which the base is unpaired). Our aim, here, is to study TDM at the gene level. MI once averaged over a whole gene is highly influenced by the second part of the equation, as numerous bases remain unpaired in all folding. It is therefore highly correlated with GC% (*r* = −0.8) and reflects more the existence of stable local structures in the genes than the relative mutability of the gene. If we define a stability index (SI) along the same line, i.e., SI = (total percentage of foldings in which the base is paired) × (the lowest −ΔG of the foldings in which the base is paired), both SI and MI correlate strongly at the gene level (*r* = 0.92), revealing that at the gene level they are not informative about mutability. We therefore defined TDMI using relative time that each base spent in an unpaired state, as described in the main text.

### Gene sequence randomisation.

To free our analysis of the various selective constraints that could have masked the selection acting to limit TDM, we performed several randomisations of gene sequences. We either fully randomised nucleotides in the sequence (conserving only GC%) or performed a synonymous codon randomisation that kept constant both gene-encoded protein sequence and gene codon bias. Using the latter randomisation, we calculated a *p*-value. For each gene, we compared the average TDMI of random genes with the average TDMI of the observed gene and defined the *p*-value as the number of random sequences having smaller TDMI than the observed sequence. A gene was said to be intrinsically robust to mutation if more than 95% of the simulations had higher TDMI values than the observed gene (i.e., *p* < 5%).

### MCU.

We calculated the MCU, a measure of codon bias, using data based on Kanaya et al. [35]: MCU = (number of major codons)/(total number of codons). Similar results were obtained using the codon adaptation index [36].

### Statistics software.

For statistics (multiple linear regressions, Student's *t-*test, chi-squared, etc.), we used the statistics software R (R Foundation for Statistical Computing, Vienna, Austria) [37].

### Operon prediction.

Operons were predicted according to the following criteria: collinear genes separated by less than 150 bases and no transcription terminator (E. Rocha, personal communication). To study the effect of gene position in the operon on TDMI, only genes in operons five genes long were used. This prevents the appearance of statistical artefacts due to differences in gene composition (GC%) as a function of operon length.

## Supporting Information

### Accession Numbers

The EntrezGenomes (http://www.ncbi.nlm.nih.gov/genomes/static/eub_g.html) accession numbers for the strains discussed in this paper are B. aphidicola APS (NC_002528), E. coli CFT073 (NC_004431), E. coli MG1655 (NC_000913), and E. coli O157:H7 (NC_002695).

## Abbreviations

CI: confidence interval
GC%: GC percent content
MCU: major codon usage
L4 site: 4-fold degenerate site
TDM: transcription-driven mutagenesis
TDMI: transcription-driven mutagenesis index
